# Neoantigen Dendritic Cell Vaccination Combined with Anti-CD38 and CpG Elicits Anti-Tumor Immunity against the Immune Checkpoint Therapy-Resistant Murine Lung Cancer Cell Line LLC1

**DOI:** 10.3390/cancers13215508

**Published:** 2021-11-02

**Authors:** Changbo Sun, Koji Nagaoka, Yukari Kobayashi, Hidewaki Nakagawa, Kazuhiro Kakimi, Jun Nakajima

**Affiliations:** 1 Department of Immunotherapeutics, The University of Tokyo Hospital, Tokyo 113-8655, Japan; sonc-sur@h.u-tokyo.ac.jp (C.S.); knagaoka@m.u-tokyo.ac.jp (K.N.); yukkoba@m.u-tokyo.ac.jp (Y.K.); 2 Department of Thoracic Surgery, The University of Tokyo Graduate School of Medicine, Tokyo 113-8655, Japan; nakajima-tho@h.u-tokyo.ac.jp; 3 Laboratory for Cancer Genomics, RIKEN Center for Integrative Medical Sciences Kanagawa Japan, Yokohama 230-0045, Japan; hidewaki@riken.jp

**Keywords:** neoantigen, DC vaccine, immunotherapy, checkpoint, combination therapy, tumor microenvironment

## Abstract

**Simple Summary:**

Despite the substantial achievements to date, a significant proportion of patients still fail to benefit from immune-checkpoint therapies (ICT). The absence of T cell infiltration and insufficient immune recognition may account for the primary resistance to immune checkpoint therapy. The present study compared the ICT response of two murine lung cancer cell line models, ASB-XIV and LLC1. ASB-XIV tumors are inflamed and are sensitive to ICT, while non-inflamed LLC1 tumors are resistant. We employed in-depth tumor analysis, including whole-exome sequencing, RNA-sequencing, and flow cytometry, to reveal the molecular mechanisms of resistance to ICT, and sought strategies to promote inflammatory/immunogenic pathway activation and inhibit immunosuppressive factors present in LLC1 tumors. An appropriate vaccination strategy combining neoantigen peptide-pulsed DC with anti-CD38 antibody can render an ICT-resistant “cold” tumor susceptible to immune rejection via a mechanism involving neutralization of regulatory T cells. Thus, the future direction of ICT is combination immunotherapy.

**Abstract:**

An important factor associated with primary resistance to immune-checkpoint therapies (ICT) is a “cold” tumor microenvironment (TME), characterized by the absence of T cell infiltration and a non-inflammatory milieu. Whole-exome and RNA sequencing to predict neoantigen expression was performed on the LLC1 cell line which forms “cold” tumors in mice. Dendritic cell (DC)-based vaccination strategies were developed using candidate neoantigen long peptides (LPs). A total of 2536 missense mutations were identified in LLC1 and of 132 candidate neoantigen short peptides, 25 were found to induce CD8^+^ T cell responses. However, they failed to inhibit LLC1 growth when incorporated into a cancer vaccine. In contrast, DCs pulsed with LPs induced CD4^+^ and CD8^+^ T cell responses and one of them, designated L82, delayed LLC1 growth in vivo. By RNA-Seq, CD38 was highly expressed by LLC1 tumor cells and, therefore, anti-CD38 antibody treatment was combined with L82-pulsed DC vaccination. This combination effectively suppressed tumor growth via a mechanism relying on decreased regulatory T cells in the tumor. This study demonstrated that an appropriate vaccination strategy combining neoantigen peptide-pulsed DC with anti-CD38 antibody can render an ICT-resistant “cold” tumor susceptible to immune rejection via a mechanism involving neutralization of regulatory T cells.

## 1. Introduction

Cancer treatment, including for lung cancer, has been radically altered by the advent of immune checkpoint therapy (ICT) over the last few years [1]. Lung cancer is the leading cause of cancer death, with an estimated 1.8 million deaths worldwide [2]. ICT was first approved for second-line or later therapy for advanced disease, then for the first-line treatment for patients whose tumors expressed PD-L1 on at least 50% of the tumor cells [3,4,5]. Currently, combination ICT-chemotherapy is approved for first-line treatment of patients with either squamous or non-squamous non-small cell lung cancer, regardless of PD-L1 expression [6]. However, many patients fail to respond to ICT, or eventually progress after initially responding [7,8]. There is. therefore, an unmet medical need to understand why some lung cancers are resistant to ICT.

A major characteristic associated with primary resistance is the so-called “cold” tumor or “T cell non-inflamed” state, where cancers lack T cell infiltration [9]. There is evidence that several factors account for this, including an absence of tumor antigens, defects in antigen presentation, insufficient T cell activation, or impaired trafficking [10]. Therefore, more effective immunotherapeutic interventions are needed to promote de novo inflammation in the non-inflamed tumor microenvironment (TME). Importantly, in addition to the above mechanisms, multiple immunosuppressive factors in the TME may contribute to resistance to ICT [11]. The complexity of cellular and molecular interactions within the immunosuppressive TME must be elucidated and manipulated to lower the risk of resistance and/or prolong the benefits of immunotherapy [12].

In the present study, we compared the ICT response of two murine lung cancer cell line models, ASB-XIV and LLC1. ASB-XIV tumors are inflamed and are sensitive to ICT, while non-inflamed LLC1 tumors are resistant. We employed in-depth tumor analysis, including whole-exome sequencing, RNA-sequencing, and flow cytometry, to reveal the molecular mechanisms of resistance to ICT, and sought strategies to promote inflammatory/immunogenic pathway activation and inhibit immunosuppressive factors present in LLC1 tumors.

## 2. Materials and Methods

### 2.1. Mice, Tumor Cells and Reagents

Six-week-old female C57BL/6N and BALB/c mice were purchased from Japan SLC (Shizuoka, Japan). All mice were kept in a specific pathogen-free environment. Lewis lung carcinoma cells (LLC1) and ASB-XIV cell lines were obtained from ATCC (CRL-1642, Manassas, VI, USA) and CLS (400120, Eppelheim, Germany), respectively, and maintained in Dulbecco’s modified Eagle’s medium (DMEM, Nacalai Tesque, Kyoto, Japan) with 10% heat-inactivated fetal bovine serum (Sigma-Aldrich, St. Louis, MI, USA), 100 µg/mL streptomycin, and 100 U/mL penicillin (FUJIFILM Wako Pure Chemical Corporation, Osaka, Japan). Class C CpG oligodeoxynucleotide (ODN2395: 5′-tcgtcgttttcggcgcgcgccg-3′) with a phosphorothioate backbone was purchased from Genscript Japan (Tokyo, Japan). A2A receptor antagonist (SCH58261) and A2B receptor antagonist (PSB1115) were obtained from Tokyo Chemical Industry (Tokyo, Japan) and R&D Systems (Minneapolis, MN, USA), respectively. Monoclonal antibodies (mAbs) specific for PD-1 (RMP1-14), CTLA-4 (9H10), CD38 (NIMR5), and CD16/32 (2.4G2) were purchased from BioXcell (Lebanon, NH, USA). APC-conjugated anti-CD103 and PE-CF594-conjugated anti-Ly6G mAbs were from BD Biosciences (Franklin Lakes, NJ, USA). FITC-conjugated anti-I-A/I-E, anti-CD3, CD19 and F4/80 mAbs, PerCP/Cy5.5-conjugated anti-CD11b, anti-CD4, APC-conjugated anti-IFN-γ, CD39, CD73 and PE/Cy7-conjugated anti-Ly6C and APC/Cy7-conjugated anti-CD8α and anti-CD45 and Pacific Blue-conjugated anti-CD38, anti-CD45 mAbs were all from BioLegend (San Diego, CA, USA). PE-conjugated anti-Foxp3 mAb was purchased from eBioscience (San Diego, CA, USA).

### 2.2. Whole-Exome Sequencing

Genomic DNA was extracted from LLC1 cells and from the tails of normal C57BL/6 mice using Allprep DNA/RNA mini kits (Qiagen, Venlo, The Netherlands), according to the manufacturer’s protocols. The genomic DNA sample was randomly fragmented to generate fragments with a base pair peak of 150 to 200 bp; adapters were ligated to both ends of the resulting fragments. The adapter-ligated templates were amplified and hybridized to the SureSelect Biotinylated RNA Library (Agilent Technologies, Santa Clara, CA, USA) for enrichment. Hybridized fragments were bound to the streptavidin beads, whereas non-hybridized fragments were washed out after 24 h. The captured library was loaded onto the HiSeq 2000 platform (Illumina, San Diego, CA, USA). After trimming adapter sequences, reads were aligned to the mm10 mouse reference sequence using the Burrows-Wheeler Aligner (BWA). Somatic variants were detected using MuTect V1.1.4. Raw data were deposited in the Sequence Read Archive (SRA) database (accession number: SRR15647454, https://trace.ncbi.nlm.nih.gov/Traces/sra/?run=SRR15647454, accessed on 20 October 2021 and SRR15647456, https://trace.ncbi.nlm.nih.gov/Traces/sra/?run=SRR15647456, accessed on 20 October 2021).

### 2.3. RNA Sequencing

Total RNA was isolated from tumor tissues using TRIzol (Thermo Fisher Scientific, Waltham, MA, USA) and RNeasy spin columns (Qiagen, Venlo, The Netherlands), according to the manufacturer’s protocols. Libraries were prepared using Illumina TruSeq Stranded mRNA Library Prep (Illumina) or NEBNext Ultra II RNA Library Prep Kit (New England Biolabs, Ipswich, MA, USA), according to the manufacturer’s protocols. The libraries were sequenced as 150 bp paired-end reads using HiSeq 2500 or NovaSeq 6000 (Illumina). The sequence reads were aligned to the mm10 reference genome using STAR V.2.7.0f. Mapped reads were counted by featureCounts V.1.6.4. Batch correction was performed using ComBat-seq from the sva package [13]. Fragments per kilobase of exon per million reads mapped (FPKM) was calculated by the formula: Y/LN × 10^9^, where Y is the number of fragments mapped to a gene; L is the length of the gene; and N is the total number of mapped reads of a sample.

Immune and stromal cell composition in tumors was estimated using mMCP-counter [14]. Raw data were deposited in the Gene Expression Omnibus (GEO) database (GSE183283, https://www.ncbi.nlm.nih.gov/geo/query/acc.cgi?acc=GSE183283, accessed on 20 October 2021).

### 2.4. Neoantigen Prediction

Somatic single nucleotide variants (SNVs) were screened from whole-exome sequencing data. The expression of tumor-specific non-synonymous variants was identified using RNA-Seq data. Mutant candidates with >1 FPKM were selected. Neoantigens were predicted and prioritized by their binding affinity to MHC class I molecules predicted by NetMHCpan 2.8. Sixty-one mutated peptides with NetMHCpan score IC_50_ < 200 nM and 71 mutated peptides with IC_50_ > 200 nM, but with a ratio of wild to corresponding mutated type peptide in NetMHCpan score > 10 were selected as candidate neoantigens. Overall, short peptides of 8 to 10 amino acid length were synthesized and long peptides (LP, 21 amino acids long with the point mutation at middle), including the sequence of immunogenic short mutated peptides, were synthesized.

### 2.5. Peptide Synthesis

Peptides were synthesized by a standard solid-phase method using Syro I (Biotage, Uppsala, Sweden). Fmoc-protected amino acid-loaded resins and Fmoc-protected amino acids were purchased from Merck (Darmstadt, Germany). Additionally, 1-[Bis(dimethylamino)methylene]-1H-1,2,3-triazolo[4,5-b]pyridinium 3-oxide hexafluorophosphate (HATU, Merck) was used in the coupling reaction. After cleavage and deprotection of peptides using reagent K (trifluoroacetic acid/phenol/thioanisole/1,2-ethanedithiol, 82.5/5/5/2.5), cold diethyl ether was added to precipitate the peptides. Sequences were confirmed by matrix-assisted laser desorption/ionization time-of-flight mass spectrometry (MALDI-TOF MS) (TOF/TOF5800, AB SCIEX, Framingham, MA, USA).

### 2.6. Neoantigen Immunization

Bone marrow-derived dendritic cells (DCs) were prepared as described previously [15]. Briefly, bone marrow cells from femurs and tibias were cultured in RPMI-1640 (Nacalai Tesque) supplemented with 10% FBS, 12.5 mM HEPES, 5 × 10^−5^ M 2-mercaptoethanol, 1 × 10^−5^ M sodium pyruvate, 1% nonessential amino acids, 100 U/mL penicillin, 100 μg/mL streptomycin, and 20 ng/mL GM-CSF (PeproTech, Rocky Hill, NJ, USA) for 8 days. DCs were stimulated with 1 µg/mL lipopolysaccharide (FUJIFILM Wako, Osaka, Japan), 10 ng/mL GM-CSF, and 10 ng/mL interleukin-4 (PeproTech) overnight and pulsed with short peptide at 1 µg/mL for 2 h. DCs were pulsed with LPs (5 µg/mL) overnight and stimulated with lipopolysaccharide (1 µg/mL), GM-CSF (10 ng/mL) and interleukin-4 (10 ng/mL) for 4 h. To immunize mice, 1 × 10^6^ DCs were subcutaneously injected into the flank.

### 2.7. Treatment of Tumor-Bearing Mice

CpG (30 µg) was injected subcutaneously into the right flank of the mice one day before tumor cell inoculation. Animals were inoculated subcutaneously into the right flank on day 0. To block CTLA-4 and/or PD-1 signaling, anti-CTLA-4 (100 μg) and/or -PD-1 mAbs (200 μg) were injected intraperitoneally on days 0, 4, and 8 and/or days 3, 6, and 9, respectively. Anti-CD38 mAb was injected intraperitoneally on days 5, 10, and 15. Tumor growth was monitored every 2–3 days with calipers, and tumor volume was calculated by the formula π/6×L1L2H, where L1 is the long diameter, L2 is the short diameter, and H is the height of the tumor.

### 2.8. Cell Preparation and Flow Cytometry

Tumors were cut into pieces and incubated in RPMI-1640 (Nacalai Tesque) supplemented with 0.2% collagenase (FUJIFILM Wako) and 2 KU/mL DNase I (Sigma-Aldrich) for 40 min at 37 °C. All material was passed through a 70 µm cell strainer to obtain single-cell suspensions. After staining dead cells using the Zombie Aqua Fixable Viability Kit (BioLegend) and blocking Fc receptors with anti-CD16/32 mAb, the cells were stained with mAbs for cell surface antigens. For intracellular cytokine staining, cells were first stimulated with 1 μg/mL corresponding peptide in the presence of 10 µg/mL brefeldin A (Sigma-Aldrich) for 4 h. After staining dead cells using the Zombie Yellow Fixable Viability Kit (BioLegend) and blocking Fcγ receptors with anti-CD16/32 mAb, cells were first stained with mAbs for surface antigens. After fixation and permeabilization using Fixation Buffer and Intracellular Staining Perm Wash Buffer (BioLegend), according to the manufacturer’s protocols, cells were then stained with APC-conjugated anti-IFN-γ antibodies. For Foxp3 assessment, cells were first stained with mAbs for cell surface antigens after staining dead cells using the Zombie Yellow Fixable Viability Kit (BioLegend) and blocking Fcγ receptors with anti-CD16/32 mAb. After fixation and permeabilization using True-Nuclear Transcription Factor Buffer Set (BioLegend), according to the manufacturer’s protocols, cells were then stained with PE-conjugated anti-Foxp3 antibodies or isotype control. Stained cells were acquired on a CytoFLEX S or Gallios flow cytometer (Beckman Coulter, Atlanta, GA, USA) and analyzed using FlowJo software V.10.6.2 (BD Biosciences). Gating strategies were shown in Figure S1.

### 2.9. Statistical Analysis

Data are presented as mean (± SD). Statistical analyses were performed with Prism software version 8.4.3 (GraphPad Software, San Diego, CA, USA). Comparisons of results were carried out by Student’s *t*-test, Fisher’s exact test, or analysis of variance with ANOVA tests for multiple comparisons.

## 3. Results

### 3.1. LLC1 Tumors Are Resistant to Immune Checkpoint Blockade

**Figure 1:**
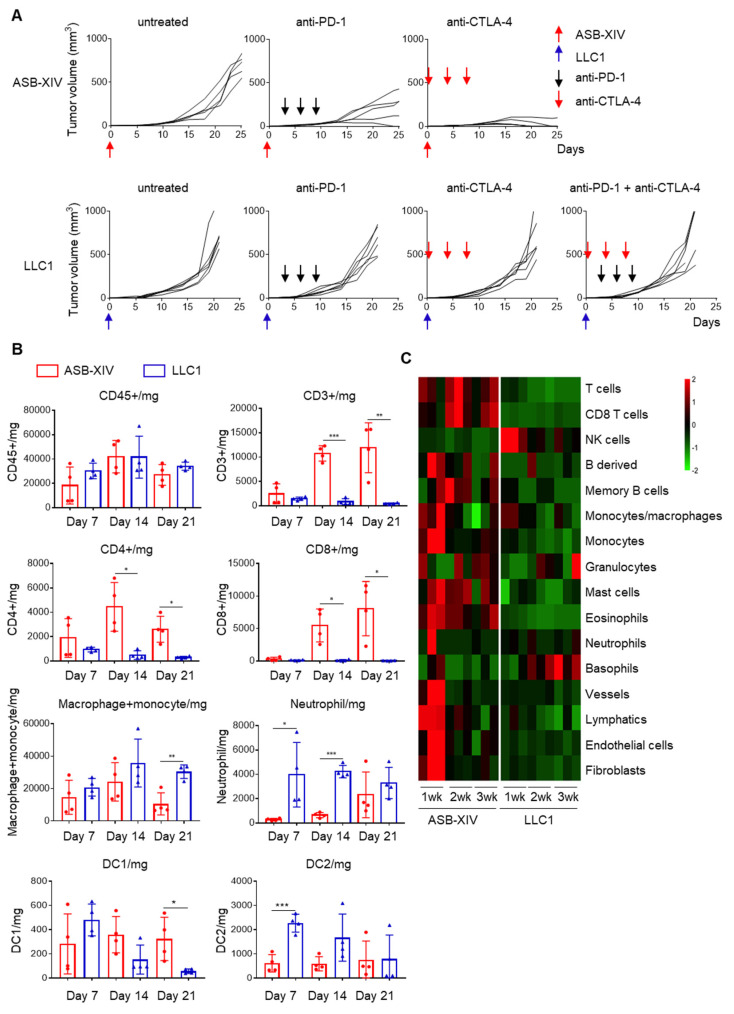
LLC1 is resistant to immune checkpoint blockades, while ASB-XIV is sensitive. (**A**) BALB/c (*n* = 5) and C57BL/6 mice (*n* = 6) were subcutaneously inoculated with 1 × 10^6^ cells of ASB-XIV and LLC1 lung cancer cells, respectively. Then, mice received anti-PD-1 (200 μg), anti-CTLA-4 mAb (100 μg), or a combination of both treatments. Anti-PD-1 was administered on days 3, 6, and 9. Anti-CTLA-4 mAb was administered on days 0, 4, and 8. (**B**) Tumor-infiltrating immune cells in ASB-XIV and LLC1 tumors (*n* = 4) were analyzed by flow cytometry on days 7, 14, and 21. (**C**) RNA expression of LLC1 tumors (*n* = 3) was evaluated on days 7, 14, and 21. The composition of tumor-infiltrating immune cells was evaluated by mMCP-counter. * *p* < 0.05, ** *p* < 0.01, *** *p* < 0.001.

BALB/c and C57BL/6 mice were subcutaneously inoculated with 1 × 10^6^ cells of the lung cancer lines ASB-XIV or LLC1, respectively, and then received anti-PD-1, anti-CTLA-4, or a combination of both antibodies. Both cell lines generated tumors growing progressively in untreated mice (Figure 1A), whereas treatment with anti-PD-1 or anti-CTLA-4 antibodies inhibited ASB-XIV tumor growth. Anti-CTLA-4 treatment was particularly effective in those tumors which were completely rejected in four out of five mice. In contrast, anti-PD-1, anti-CTLA-4, or even a combination of both failed to make any impact on LLC1 tumor growth (Figure 1A). These results document that ASB-XIV is sensitive to ICT whereas LLC1 is highly resistant.

To identify factors influencing the sensitivity of these two cell lines to ICT, tumor-infiltrating cells were isolated and analyzed by flow cytometry. Tumors were harvested on days 7, 14, and 21 and enzymatically dissociated into a single-cell suspension. As shown in Figure 1B, a similar number of CD45^+^ tumor-infiltrating cells was obtained from both ASB-XIV and LLC1 tumors. However, the tumor-infiltrating immune cells were quite different: many CD4^+^ and CD8^+^ T cells infiltrated ASB-XIV and increased from day 7 to day 14, while only a few T cells infiltrated the LLC1 tumors and decreased from day 7 to day 14. In contrast, more neutrophils, macrophages, and monocytes were detected in LLC1 tumors than ASB-XIV and outnumbered the T cells (Figure 1B).

To compare the TME of tumors caused by these two lung cancer cell lines, total RNA was extracted from day 7, 14, and 21 tumors and subjected to RNA-Seq analysis. The infiltration of immune cells was evaluated using the mMCP-counter [14]. The expression of many immune signature genes was lower in LLC1 than ASB-XIV tumors (Figure 1C). Taken together with the flow cytometry data, we conclude that ASB-XIV is an inflamed or “hot” tumor, while LLC1 is a non-inflamed or “cold” tumor.

### 3.2. Identification of Mutation-Associated Neoantigens in LLC1 Tumors

To convert cold LLC1 tumors into hot tumors, we investigated LLC1-specific T cell responses induced by mutation-associated neoantigens. We first performed whole-exome sequencing of LLC1 cells and identified 2536 missense mutations. RNA-Seq was performed to determine whether products of these mutated genes were present in LLC1 cells. We identified 856 expressed missense mutated genes with FPKM > 1. These mutated genes were translated in silico, and 8-, 9-, and 10-mer peptides containing the mutated amino acid were predicted. NetMHCpan was then employed to estimate their binding to H-2K^b^ or D^b^ molecules. Of these, we selected 61 with an estimated MHC binding affinity IC_50_ < 200 nM and 71 with IC_50_ > 200 nM, but for which the ratio between the binding affinity of the corresponding wild-type and the mutated peptide was >10. We then synthesized these 132 peptides (Table S1) and tested them for immunogenicity in C57BL/6 mice.

### 3.3. Immunogenicity of Predicted Neoantigen Peptides

**Figure 2:**
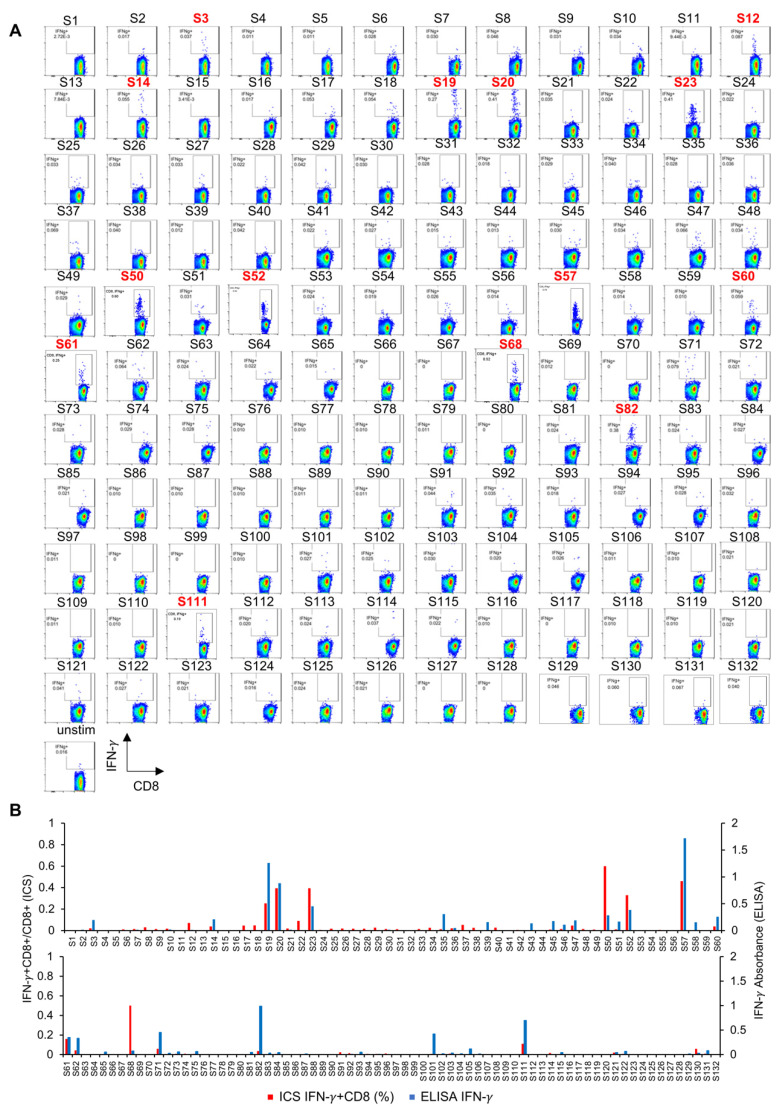
Identification of mutation-associated neoantigens in LLC1 tumors. (**A**) C57BL/6 mice (*n* = 3 or 4) were immunized with 1 × 10^6^ dendritic cells (DC) pulsed with these neoantigen short peptides (Table S1). After two rounds of DC immunization at a 2-week interval, spleen cells from immunized mice were harvested, pooled, and examined for the reactivity to the immunized peptide by ex vivo intracellular cytokine staining (ICS). By ex vivo ICS analysis, 14 peptides (highlighted in red numbers) induced peptide-specific IFN-γ production of CD8^+^ T cells. (**B**) Spleen cells were also cultured with immunized peptides for 5 days and restimulated with corresponding peptides. After in vitro expansion culture, IFN-γ production was examined by ELISA. Red bars and blue bars represent the results of ex vivo ICS and ELISA after in vitro culture, respectively. Overall, 25 mutated peptides displayed immunogenicity by DC vaccination.

C57BL/6 mice were immunized with DCs pulsed with these neoantigen peptides in the form of short peptides (8- to 10-mers). After two rounds of DC immunization (2 weeks), spleen cells were harvested and tested for reactivity to the immunizing peptide by ex vivo intracellular cytokine staining (ICS) (Figure 2A,B). Fourteen peptides were found to induce peptide-specific IFN-γ-producing CD8^+^ T cells. Spleen cells were also cultured with immunizing peptides for 5 days and restimulated with the corresponding peptides. IFN-γ production was measured by ELISA (Figure 2B). After in vitro expansion culture, IFN-γ production was detected in 23 peptide-stimulated cultures. Altogether, 25 mutated peptides were identified that displayed immunogenicity on DC vaccination.

**Figure 3:**
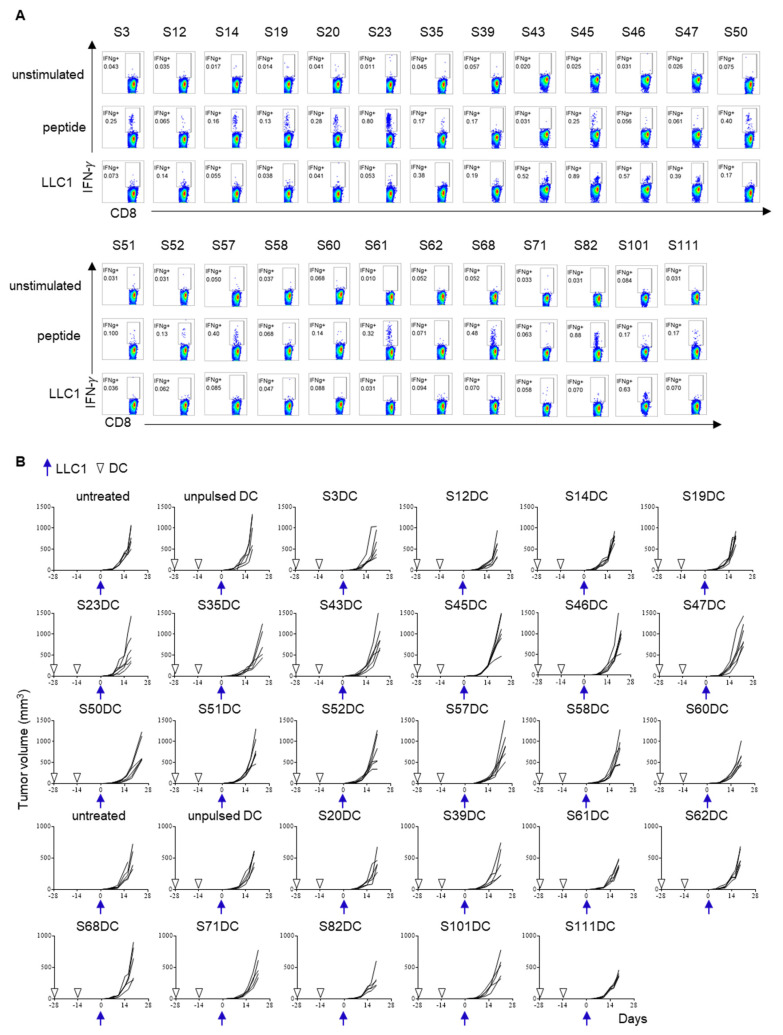
The immunogenicity and anti-tumor activity of 25 short mutated peptides. DCs were pulsed with one of these 25 mutated peptides. Groups (8–10 mice per group) were immunized twice with each peptide-pulsed DC vaccine (1 × 10^6^) at a 2-week interval. Three or four mice were used for immunogenicity assessment (**A**), and 5–6 mice were used for the LLC1 cells challenge (**B**). IFN-γ production in response to immunized peptide and LLC1 cells was evaluated. In addition, tumor growth was measured after LLC1 cells (1 × 10^6^) were challenged in these immunized mice.

The anti-tumor activity of these neoantigen vaccines was evaluated in prophylactic DC vaccination experiments. DCs were separately pulsed with each one of these 25 mutated peptides and groups of 8–10 mice were immunized twice with each at a 2-week interval. As shown in Figure 3A, peptide-specific CD8^+^-T cell responses were detected in splenocytes by the production of IFN-γ. Vaccinated mice were challenged with LLC1 cells, but no short peptide-pulsed DC vaccine significantly inhibited tumor growth (Figure 3B). These data indicate that vaccination with DCs pulsed with a single neoantigen short peptide alone was not sufficient to prevent LLC1 tumor formation.

### 3.4. Improved Immunogenicity of Neoantigen Peptide-Pulsed DC Vaccine in the LP Format

**Figure 4:**
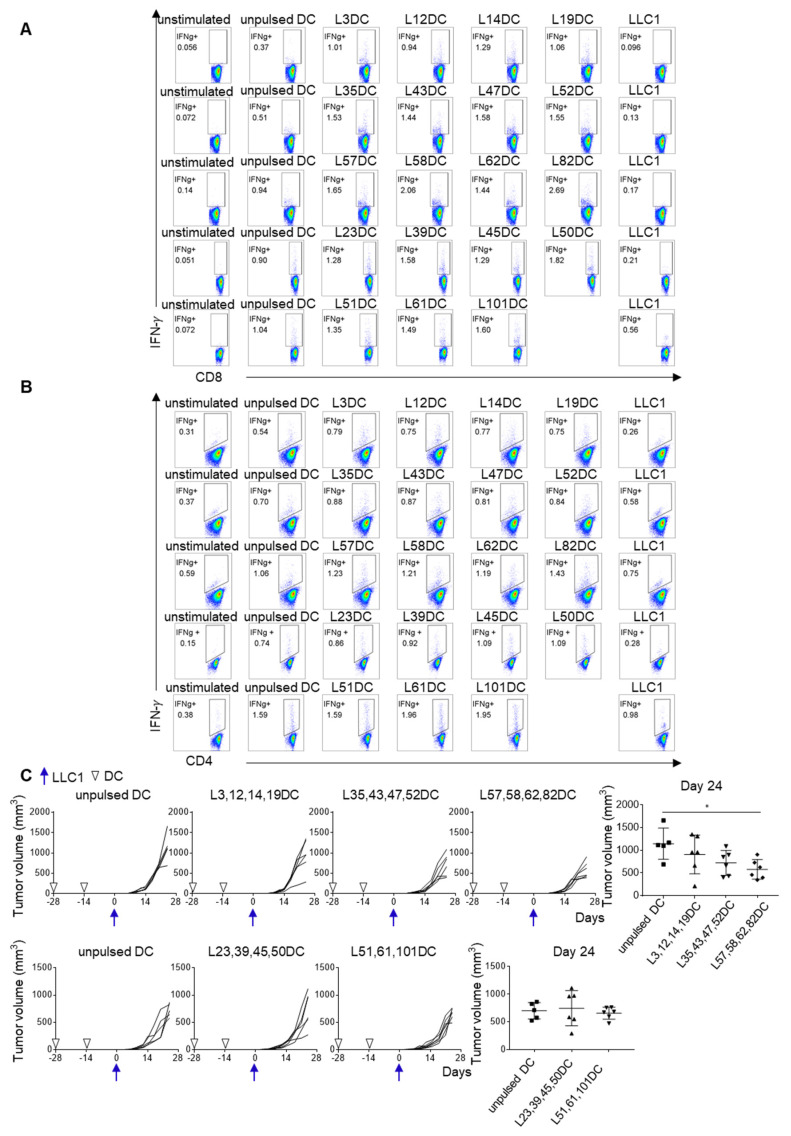
Improved immunogenicity of neoantigen-peptide pulsed DC vaccine in the LP format. Nineteen LPs (21-mer), incorporating corresponding immunogenic 25 short mutated peptide sequences, were synthesized (Table S2). Three or four different LP-pulsed DCs were put together to produce five mixtures of DC vaccines: group A consists of L3, L12, L14, and L19 LPs; group B consists of L35, L43, L47, and L52 LPs; group C consists of L57, L58, L62, and L82 LPs; group D consists of L23, L39, L45, and L50 LPs; and group E consists of L51, L61, and L101 LPs. Mice were immunized twice at a 2-week interval. Two weeks after the last immunization, spleen cells were harvested and examined for the reactivity to the immunized LP by ex vivo ICS. IFN-γ production by CD8^+^ T cells (**A**) and CD4^+^ T cells (**B**) were evaluated by ICS. (**C**) Two weeks after the last immunization, LLC1 cells (1 × 10^6^) were subcutaneously inoculated (5 or 6 mice per group). Tumor growth was measured twice a week. * *p* < 0.05.

To further investigate the efficacy of the 25 candidate neoantigens, 19 LPs (21-mer), which incorporated the corresponding immunogenic 25 short peptide sequences, were synthesized and pulsed onto DCs (Table S2). Although CD4^+^ and CD8^+^ spleen cells from mice vaccinated with these LP-pulsed DCs produced IFN-γ in response to the immunizing peptide (Figure 4A,B), their response to LLC1 tumor cells was barely detectable. The anti-tumor activity of these LP neoantigen-pulsed DCs peptides was also evaluated by prophylactic DC vaccination for which DCs pulsed with each of 25 LPs were mixed (three or four different LP-pulsed DCs in one vaccine) to yield five different vaccine products that were used to immunize mice twice at a 2-week interval. Two weeks after the final immunization, vaccinated mice were challenged with LLC1 cells. LLC1 tumor growth was partially suppressed in some vaccinated mice. The most active mixture included L57, L58, L62, and L82 LPs (*p* = 0.042) (Figure 4C).

### 3.5. CpG Converts Cold Tumors to Hot

**Figure 5:**
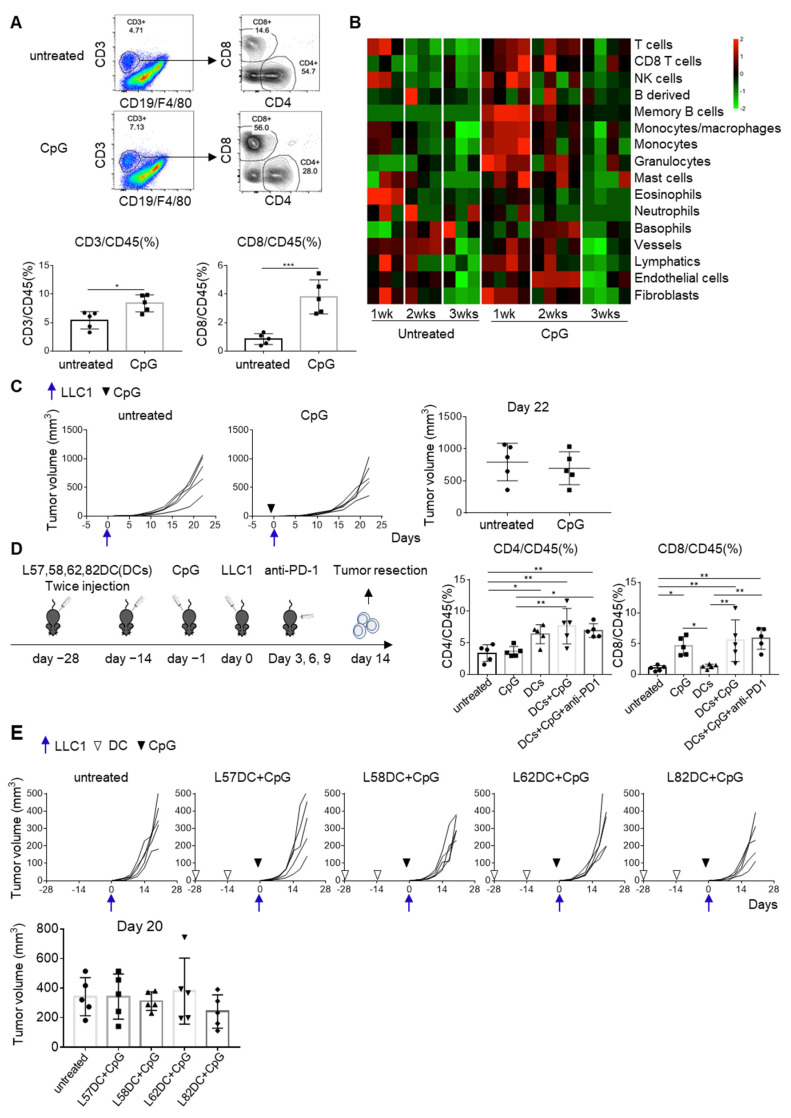
CpG treatment turned cold tumors into hot. (**A**) CpG (30μg) was subcutaneously administered to C57BL/6 mice (*n* = 5) one day before 1×10^6^ LLC1 cells inoculation. Tumors were harvested on day 14 and tumor-infiltrating lymphocytes were analyzed by flow cytometry. (**B**) RNAs were extracted from days 7, 14, and 21 tumors and subjected to RNA-Seq. The composition of tumor-infiltrating immune cells was analyzed using the mMCP-counter. (**C**) C57BL/6 mice (*n* = 5) were either treated with 30μg CpG or left untreated, and LLC1 tumor growth was evaluated. (**D**) C57BL/6 mice were divided into five groups (five mice per group): (1) untreated; (2) CpG treatment; (3) the mixture of L57, L58, L62, and L82 LP-pulsed DC (1 × 10^6^ each) vaccination; (4) the combination of DC vaccination and CpG; and (5) the triple combination of DC vaccination, CpG and anti-PD-1 mAb (200μg). Mice received DC vaccination on days −28 and −14. CpG was administered on day −1. Mice received anti-PD-1 mAb on days 3, 6, and 9 after the LLC1 challenge (1 × 10^6^). On day 14, tumors were resected and subjected to the analysis of tumor-infiltrating cells. CD4/CD45 (%): untreated vs. DCs (*p* = 0.0316), untreated vs. DCs+CpG (*p* = 0.0021), untreated vs. DCs+CpG+anti-PD-1 (*p* = 0.0096), CpG vs. DCs+CpG (*p* = 0.0035), CpG vs. DCs+CpG+anti-PD-1 (*p* = 0.0161); CD8/CD45 (%): untreated vs. CpG (*p* = 0.0199), untreated vs. DCs+CpG (*p* = 0.0038), untreated vs. DCs+CpG+anti-PD-1 (*p* = 0.0018), CpG vs. DCs (*p* = 0.0336), DCs vs. DCs+CpG (*p* = 0.0066), DCs vs. DCs+CpG+anti-PD-1 (*p* = 0.0032). (**E**) Tumor growth was compared in these five groups. * *p* < 0.05, ** *p* < 0.01, *** *p* < 0.001.

In an attempt to improve DC vaccine efficiency, CpG (30 μg) was administered subcutaneously to C57BL/6 mice the day before tumor inoculation. Tumors were harvested on day 14 and tumor-infiltrating lymphocytes (TILs) were analyzed by flow cytometry. More TILs were obtained from mice receiving CpG than from untreated mice. CD3^+^ T cell, especially CD8^+^ T cell, infiltration was increased in tumors from CpG-treated mice (Figure 5A). Consistently, RNA-Seq indicated a massive infiltration of immune cells, including T cells, B cells, monocyte/macrophages and granulocytes, into the tumor (Figure 5B). Nonetheless, CpG treatment alone was insufficient to inhibit LLC1 tumor growth (Figure 5C). As shown in Figure S2A, increased expression of effector molecules was detected in CpG treated mice; however, the expressions of immune checkpoint molecules were lower in CpG mice than untreated mice. In contrast, the expressions of immunosuppressive molecules, such as molecules in the adenosine pathway, arginase, TGFβ and IL10, were higher in CpG treated mice than untreated mice (Figure S2B). Therefore, we concluded that the induction of the immunosuppressive microenvironment was responsible for the insufficient anti-tumor activity in the presence of T cell infiltration. More T cell expansion that overcomes the immunosuppressive microenvironment is required. Therefore, we combined CpG treatment with DC vaccination. The mixture of L57, L58, L62, and L82 LP-pulsed DC vaccine increased CD4^+^ T cells, while CpG increased CD8^+^ T cells in the TME 14 days after tumor inoculation (Figure 5D). Mice then received individual L57, L58, L62, and L82 LP-pulsed DC vaccines together with CpG. Of these, DCs pulsed with L82 plus CpG partly delayed LLC1 growth in vivo, although this did not achieve statistical significance (Figure 5E).

### 3.6. The Immunosuppressive Microenvironment Is Associated with CD38 Expression

**Figure 6:**
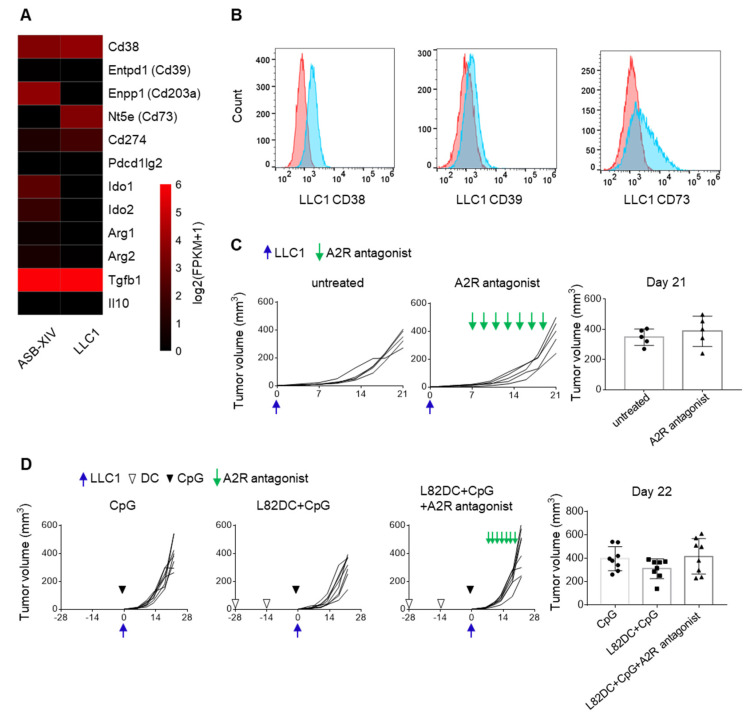
The adenosine pathway in the LLC1 tumor microenvironment. (**A**) RNA was extracted from ASB-XIV and LLC1 tumor cells and subjected to RNA-Seq analysis. The mRNA expressions of immunosuppressive molecules in these two cell lines were shown. (**B**) The expressions of CD38, CD39, and CD73 on LLC1 were examined by flow cytometry in vitro. (**C**) C57BL/6 mice (*n* = 5) received a subcutaneous injection of 5 × 10^5^ LLC1. The combination of 2 mg/Kg A2A and 1 mg/Kg A2B adenosine receptor antagonists (SCH 58261 and PSB 1115) was administered on days 7, 9, 11, 13, 15, 17, and 19. (**D**) LLC1 tumor growth was compared in mice (*n* = 8) that received 30μg CpG monotherapy, L82 LP-pulsed DC vaccination (1 × 10^6^), and CpG, or the triple combination of DC vaccination, CpG, and A2R antagonists. DC vaccines were prophylactically administered twice at a 2-week interval.

To further increase the efficacy of neoantigen DC vaccines, the expression of immunosuppressive molecules in LLC1 cells were screened by RNA-Seq. As shown in Figure 6A, Cd38 and Nt5e (CD73) mRNAs were highly expressed in LLC1 cells. Furthermore, the expression of molecules associated with the adenosine pathway, namely, CD38, CD39 and CD73, were detected on the LLC1 cell surface by flow cytometry (Figure 6B). Therefore, A2A and A2B adenosine receptor antagonists (SCH 58261 and PSB 1115) were combined with prophylactic L82-pulsed DC vaccination and CpG. However, L82-pulsed DC vaccination together with CpG or in combination with A2R antagonists failed to suppress LLC1 tumor growth (Figure 6C,D).

**Figure 7:**
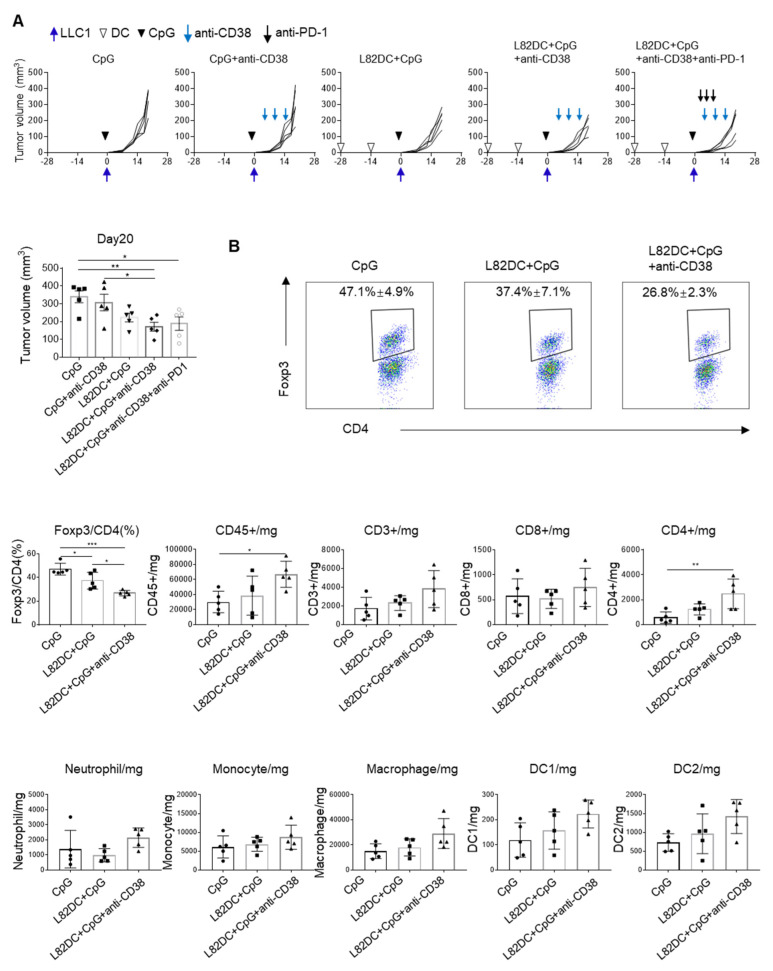
The triple combination of DC vaccination, CpG and anti-CD38 mAb treatment inhibited LLC1 tumor growth. (**A**) C57BL/6 mice were divided into five groups (five mice per group): (1) 30μg CpG treatment; (2) CpG plus 250μg anti-CD38 mAb; (3) 1×10^6^ L82 LP-pulsed DC vaccination with CpG; (4) the triple combination of L82 LP-pulsed DC vaccination, CpG, and anti-CD38 mAb; and (5) the quadruple combination of DC, CpG, anti-CD38 mAb, and 200μg of anti-PD-1 mAb. LLC1 cells (5 × 10^5^) were subcutaneously inoculated and tumor growth was monitored. (**B**) On day 14, tumor-infiltrating cells were extracted from these mice (*n* = 5) and subjected to flow cytometry. * *p* < 0.05, ** *p* < 0.01, *** *p* < 0.001.

In addition to the adenosine pathway, CD38 is also involved regulatory T cells (Tregs). Tregs were reduced in the tumor when LLC1-bearing mice were treated with anti-CD38 (Figure S3). In contrast, the combination of anti-CD38 antibody, L82-pulsed DC vaccine, and CpG suppressed LLC1 tumor growth (*p* = 0.019), although neither anti-CD38 mAb nor L82-pulsed DC monotherapy alone could do so (Figure 7A). We also tested a combination of anti-CD38, L82-pulsed DC vaccine with CpG, and anti-PD-1 mAb but this did not result in any enhancement of the anti-tumor activity of the treatment. On day 14 LLC1 tumors, we found that the combination with DC vaccine and anti-CD38 decreased the amount of intra-tumoral regulatory T cells (Tregs) (*p* = 0.0001) (Figure 7B), while there was no significant difference in CD8^+^ T cells in the treatment groups.

## 4. Discussion

Immune “cold” tumors or “T cell non-inflamed” tumors are characterized by their primary resistance to ICT, despite the success of this therapeutic approach in a variety of cancers [16,17]. This was the reason why single-agent anti-PD-1, or anti-CTLA-4 antibody treatment or both together, failed to inhibit the growth of LLC1; T cells failed to infiltrate the tumor. In contrast, the immunotherapy-sensitive, T cell-inflamed ASB-XIV exhibited robust T cell infiltration. Thus, new strategies for combination therapies, including priming immune responses by neoantigen vaccines, increasing the infiltration of T cells by CpG, and modulating the immunosuppressive environment by anti-CD38 mAb, were all required to achieve an effective anti-tumor response against immunologically cold LLC1 tumors in our model.

T cells that target tumor neoantigens arising from cancer mutations are major mediators of effective cancer immunotherapies [18]. The lack of T cell priming in non-T cell-inflamed tumors may be one critical feature associated with failed anti-tumor immunity [19]. This suggests that primary resistance might necessitate the use of a neoantigen vaccine that enhances T cell responses and cancels out suppressive effects [20]. DCs are potent antigen-presenting cells to induce CD4^+^ and CD8^+^ T cell responses [21,22]. Therefore, we applied a DC-based vaccine for neoantigen immunotherapy. In our study, only a very small percentage of mutations resulted in peptides eliciting specific CD8^+^ T cell immune responses on testing a large number of predicted short peptides, consistent with reports in other preclinical models and clinical studies [23,24,25]. However, even the short peptides that did stimulate CD8^+^ T cell responses did not result in the induction of regression of the immunologically cold LLC1 tumor. Consistent with this, a recent study reported that CD8^+^ TILs specific for mRiok1 (the same sequence as the immunogenic short peptide S50 in our study) were present in LLC1 tumors and were expanded after anti-PD-1 or anti-CTLA-4 treatment. Nonetheless, as in our model, a combination of prophylactic mRiok1 vaccination, anti-PD-1, and anti-CTLA-4 also failed to induce a protective anti-tumor response [26].

Given the heterogeneity of solid tumors and the presence of multiple antigens, it will probably be necessary to target multiple neoantigens to increase the likelihood of an effective anti-tumor immune response [27]. Consequently, our study showed that vaccines consisting of DCs pulsed with multiple immunogenic LPs were more effective, whereas those consisting of only a single LP could not control LLC1 tumor growth. The greater efficacy of long-versus-short peptides may be at least partly attributed to the fact that LP-pulsed DC induced not only CD8^+^ T cells, but also CD4^+^ T cell responses. The latter are thought to play an important “helper” role in vaccine-induced anti-tumor cytotoxic responses [28]. We also noted that the LP sequences described in a previous report as being effective did not suppress LLC1 tumor growth in our study [29].

It has been reported that Toll-like receptor (TLR) agonists engender several changes in the tumor environment that increase responsiveness to PD-1 blockade [30]. TLR9 agonist CpG has been reported to enhance infiltration of CD8^+^ T cells in the tumor [31]. In our study, CpG significantly enhanced the infiltration of CD8^+^ T cells and also decreased neutrophil and monocyte-macrophage infiltration into LLC1 tumors. Nonetheless, LLC1 still failed to respond to anti-PD-1 treatment after CpG injection. Importantly, several of our LP-pulsed DC vaccines plus CpG increased CD4^+^ and CD8^+^ T cell infiltration, but nonetheless, only slightly delayed tumor growth. These results suggest that the nature of the immunosuppressive TME, including the immune cells, needs to be elucidated for each tumor [32].

Deep-immunophenotyping of the TME may reveal the nature of the immunosuppressive microenvironment and assist in developing new combination therapeutic strategies [33]. Here, we found that molecular and gene signatures indicated that CD38 was highly expressed in LLC1. CD38 upregulation, which inhibits CD8^+^ T cell function in the TME via adenosine receptor signaling, has been reported to be involved in acquired PD-1/PD-L1 resistance [34]. Although CD38 plays an important role in converting nicotinamide adenine dinucleotide (NAD+) to adenosine, adenosine receptor antagonists did not suppress LLC1 tumor growth in our model. In contrast, CD38 blockade decreased the presence of Tregs in the LLC1 tumor microenvironment. This is in line with the findings of others that extracellular NAD+ accumulation owing to CD38 blockade decreased Tregs in the tumor [35]. Finally, we found that the combination of CpG and CD38 blockade together was able to inhibit the proliferation of LLC1 in L82-pulsed DC-immunized mice.

## 5. Conclusions

Our study describes a combinatorial approach to overcome the lack of a pre-existing immune response and ultimately convert a cold tumor into a hot tumor by increasing CD8^+^ and CD4^+^ T cell infiltration and by enhancing T cell responses by neutralization of suppressive signals in the TME.

## Data Availability

Data are deposited on the Sequence Read Archive (SRA) database (accession number: SRR15647454 and SRR15647456) and the Gene Expression Omnibus (GEO) database (GSE183283).

## Supplementary Materials

The following are available online at https://www.mdpi.com/article/10.3390/cancers13215508/s1: Figure S1: Gating strategy of flow cytometry; Figure S2: Expression of genes related to T cells and immune suppression after CpG treatment; Figure S3: Anti-CD38mAb treatment decreased the amount of intra-tumoral regulatory T cells; Table S1: The list of candidate neoantigen peptides; Table S2: The list of peptides for LP DC vaccination.

## References

[1] Pardoll D.M. (2012). The blockade of immune checkpoints in cancer immunotherapy. Nat. Rev. Cancer.

[2] Sung H., Ferlay J., Siegel R.L., Laversanne M., Soerjomataram I., Jemal A., Bray F. (2021). Global Cancer Statistics 2020: GLOBOCAN Estimates of Incidence and Mortality Worldwide for 36 Cancers in 185 Countries. CA Cancer J. Clin..

[3] Brahmer J., Reckamp K.L., Baas P., Crino L., Eberhardt W.E., Poddubskaya E., Antonia S., Pluzanski A., Vokes E.E., Holgado E. (2015). Nivolumab versus Docetaxel in Advanced Squamous-Cell Non-Small-Cell Lung Cancer. N. Engl. J. Med..

[4] Borghaei H., Paz-Ares L., Horn L., Spigel D.R., Steins M., Ready N.E., Chow L.Q., Vokes E.E., Felip E., Holgado E. (2015). Nivolumab versus Docetaxel in Advanced Nonsquamous Non-Small-Cell Lung Cancer. N. Engl. J. Med..

[5] Reck M., Rodriguez-Abreu D., Robinson A.G., Hui R., Csoszi T., Fulop A., Gottfried M., Peled N., Tafreshi A., Cuffe S. (2016). Pembrolizumab versus Chemotherapy for PD-L1-Positive Non-Small-Cell Lung Cancer. N. Engl. J. Med..

[6] Ettinger D.S., Wood D.E., Aisner D.L., Akerley W., Bauman J.R., Bharat A., Bruno D.S., Chang J.Y., Chirieac L.R., D’Amico T.A. (2021). NCCN Guidelines Insights: Non-Small Cell Lung Cancer, Version 2.2021. J. Natl. Compr. Cancer Netw..

[7] Sharma P., Hu-Lieskovan S., Wargo J.A., Ribas A. (2017). Primary, Adaptive, and Acquired Resistance to Cancer Immunotherapy. Cell.

[8] Doroshow D.B., Sanmamed M.F., Hastings K., Politi K., Rimm D.L., Chen L., Melero I., Schalper K.A., Herbst R.S. (2019). Immunotherapy in Non-Small Cell Lung Cancer: Facts and Hopes. Clin. Cancer Res..

[9] Bonaventura P., Shekarian T., Alcazer V., Valladeau-Guilemond J., Valsesia-Wittmann S., Amigorena S., Caux C., Depil S. (2019). Cold Tumors: A Therapeutic Challenge for Immunotherapy. Front. Immunol..

[10] Chen D.S., Mellman I. (2013). Oncology meets immunology: The cancer-immunity cycle. Immunity.

[11] Binnewies M., Roberts E.W., Kersten K., Chan V., Fearon D.F., Merad M., Coussens L.M., Gabrilovich D.I., Ostrand-Rosenberg S., Hedrick C.C. (2018). Understanding the tumor immune microenvironment (TIME) for effective therapy. Nat. Med..

[12] Boyero L., Sanchez-Gastaldo A., Alonso M., Noguera-Ucles J.F., Molina-Pinelo S., Bernabe-Caro R. (2020). Primary and Acquired Resistance to Immunotherapy in Lung Cancer: Unveiling the Mechanisms Underlying of Immune Checkpoint Blockade Therapy. Cancers.

[13] Zhang Y., Parmigiani G., Johnson W.E. (2020). ComBat-seq: Batch effect adjustment for RNA-seq count data. NAR Genom. Bioinform..

[14] Petitprez F., Levy S., Sun C.M., Meylan M., Linhard C., Becht E., Elarouci N., Tavel D., Roumenina L.T., Ayadi M. (2020). The murine Microenvironment Cell Population counter method to estimate abundance of tissue-infiltrating immune and stromal cell populations in murine samples using gene expression. Genome Med..

[15] Hirano K., Hosoi A., Matsushita H., Iino T., Ueha S., Matsushima K., Seto Y., Kakimi K. (2015). The nitric oxide radical scavenger carboxy-PTIO reduces the immunosuppressive activity of myeloid-derived suppressor cells and potentiates the antitumor activity of adoptive cytotoxic T lymphocyte immunotherapy. Oncoimmunology.

[16] Galon J., Bruni D. (2019). Approaches to treat immune hot, altered and cold tumours with combination immunotherapies. Nat. Rev. Drug Discov..

[17] Ascierto P.A., Bifulco C., Ciardiello F., Demaria S., Emens L.A., Ferris R., Formenti S.C., Galon J., Khleif S.N., Kirchhoff T. (2021). Perspectives in immunotherapy: Meeting report from the immunotherapy bridge (December 2nd–3rd, 2020, Italy). J. Transl. Med..

[18] Tran E., Robbins P.F., Rosenberg S.A. (2017). ‘Final common pathway’ of human cancer immunotherapy: Targeting random somatic mutations. Nat. Immunol..

[19] Chen D.S., Mellman I. (2017). Elements of cancer immunity and the cancer-immune set point. Nature.

[20] Whiteside T.L., Demaria S., Rodriguez-Ruiz M.E., Zarour H.M., Melero I. (2016). Emerging Opportunities and Challenges in Cancer Immunotherapy. Clin. Cancer Res..

[21] Harari A., Graciotti M., Bassani-Sternberg M., Kandalaft L.E. (2020). Antitumour dendritic cell vaccination in a priming and boosting approach. Nat. Rev. Drug Discov..

[22] Wang Y., Xiang Y., Xin V.W., Wang X.W., Peng X.C., Liu X.Q., Wang D., Li N., Cheng J.T., Lyv Y.N. (2020). Dendritic cell biology and its role in tumor immunotherapy. J. Hematol. Oncol..

[23] Kreiter S., Vormehr M., van de Roemer N., Diken M., Lower M., Diekmann J., Boegel S., Schrors B., Vascotto F., Castle J.C. (2015). Erratum: Mutant MHC class II epitopes drive therapeutic immune responses to cancer. Nature.

[24] Tran E., Ahmadzadeh M., Lu Y.C., Gros A., Turcotte S., Robbins P.F., Gartner J.J., Zheng Z., Li Y.F., Ray S. (2015). Immunogenicity of somatic mutations in human gastrointestinal cancers. Science.

[25] Zhang R., Yuan F., Shu Y., Tian Y., Zhou B., Yi L., Zhang X., Ding Z., Xu H., Yang L. (2020). Personalized neoantigen-pulsed dendritic cell vaccines show superior immunogenicity to neoantigen-adjuvant vaccines in mouse tumor models. Cancer Immunol. Immunother..

[26] Li S., Simoni Y., Zhuang S., Gabel A., Ma S., Chee J., Islas L., Cessna A., Creaney J., Bradley R.K. (2021). Characterization of neoantigen-specific T cells in cancer resistant to immune checkpoint therapies. Proc. Natl. Acad. Sci. USA.

[27] Saxena M., van der Burg S.H., Melief C.J.M., Bhardwaj N. (2021). Therapeutic cancer vaccines. Nat. Rev. Cancer.

[28] Blass E., Ott P.A. (2021). Advances in the development of personalized neoantigen-based therapeutic cancer vaccines. Nat. Rev. Clin. Oncol..

[29] Chen T., Hu R., Wan Y., Sun F., Wang Z., Yue J., Chen J., Han G., Wei G., Dong Z. (2020). Comprehensive mutanome analysis of Lewis lung cancer reveals immunogenic neoantigens for therapeutic vaccines. Biochem. Biophys. Res. Commun..

[30] Farooq M., Batool M., Kim M.S., Choi S. (2021). Toll-Like Receptors as a Therapeutic Target in the Era of Immunotherapies. Front. Cell Dev. Biol..

[31] Wang S., Campos J., Gallotta M., Gong M., Crain C., Naik E., Coffman R.L., Guiducci C. (2016). Intratumoral injection of a CpG oligonucleotide reverts resistance to PD-1 blockade by expanding multifunctional CD8+ T cells. Proc. Natl. Acad. Sci. USA.

[32] Duan Q., Zhang H., Zheng J., Zhang L. (2020). Turning Cold into Hot: Firing up the Tumor Microenvironment. Trends Cancer.

[33] Murciano-Goroff Y.R., Warner A.B., Wolchok J.D. (2020). The future of cancer immunotherapy: Microenvironment-targeting combinations. Cell Res..

[34] Chen L., Diao L., Yang Y., Yi X., Rodriguez B.L., Li Y., Villalobos P.A., Cascone T., Liu X., Tan L. (2018). CD38-Mediated Immunosuppression as a Mechanism of Tumor Cell Escape from PD-1/PD-L1 Blockade. Cancer Discov..

[35] Hubert S., Rissiek B., Klages K., Huehn J., Sparwasser T., Haag F., Koch-Nolte F., Boyer O., Seman M., Adriouch S. (2010). Extracellular NAD+ shapes the Foxp3+ regulatory T cell compartment through the ART2-P2X7 pathway. J. Exp. Med..

