# Supplementary material for: Neoantigen Dendritic Cell Vaccination Combined with Anti-CD38 and CpG Elicits Anti-Tumor Immunity against the Immune Checkpoint Therapy-Resistant Murine Lung Cancer Cell Line LLC1
Source: Cancers (Basel). 2021 Nov 2;13(21):5508. doi: 10.3390/cancers13215508 (PMC8583214; doi:10.3390/cancers13215508)
Supplement: Supplementary file 1 [file cancers-13-05508-s001.zip › Supplementary Figures.pdf]

Gating strategy for CD4+ and CD8+ T cells

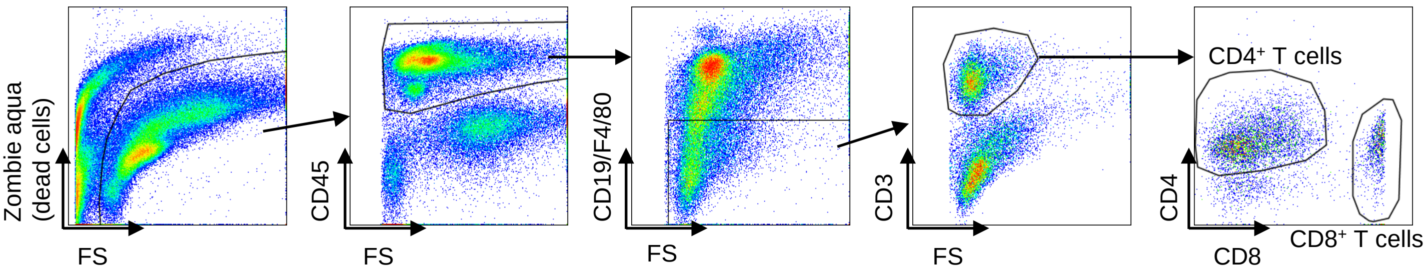

Gating strategy for macrophages, monocytes, neutrophils and dendritic cells (DC)

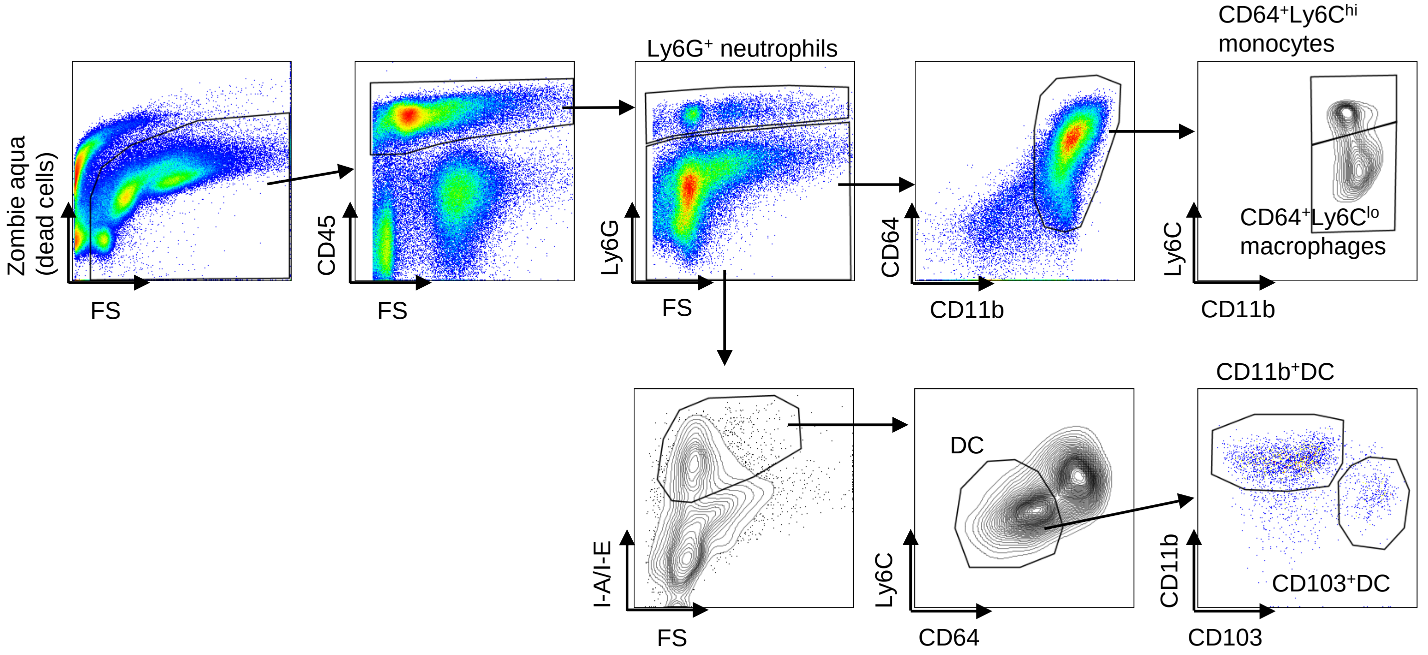

Supplementary Figure S1. Gating strategy of flow cytometry.

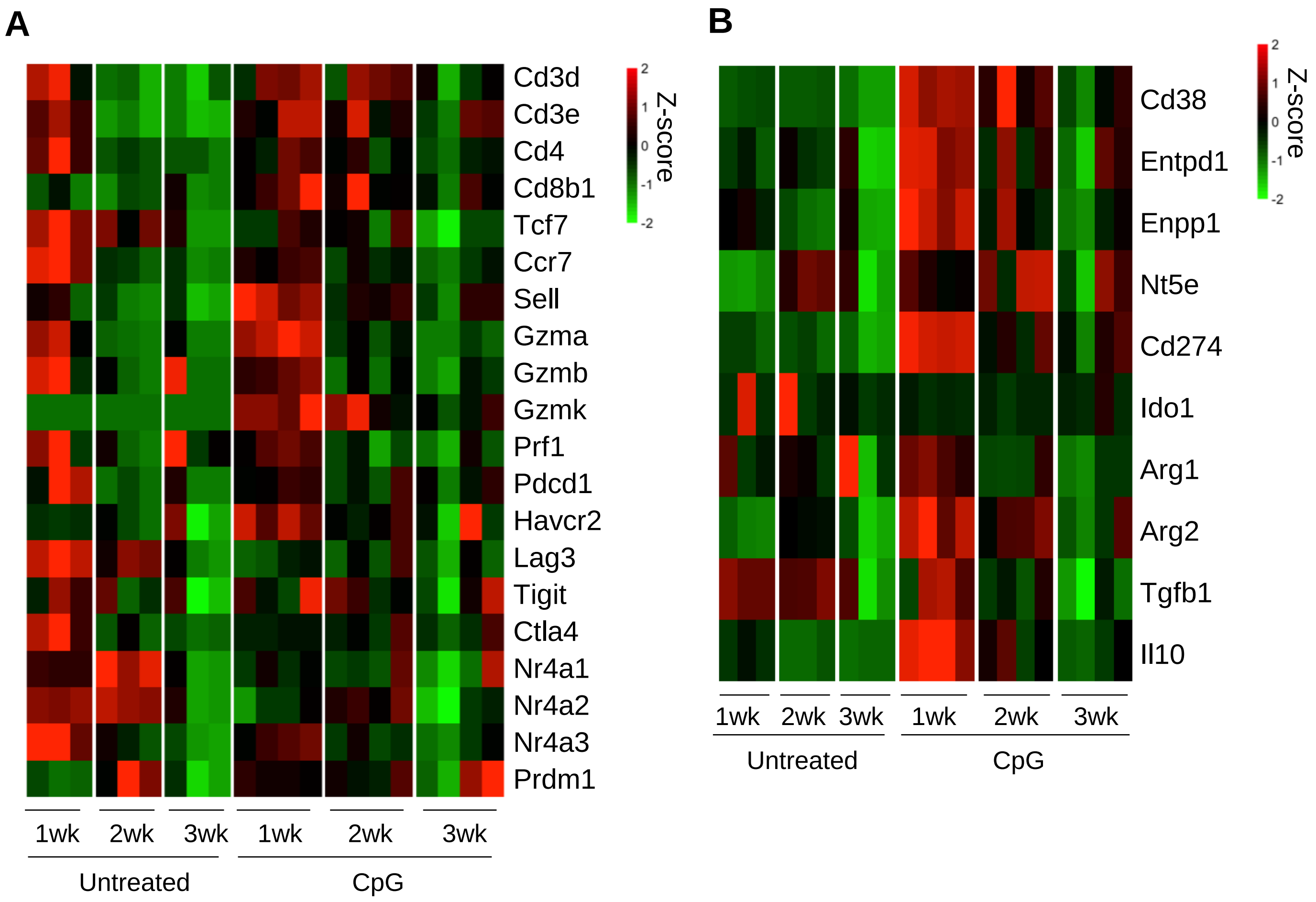

Supplementary Figure S2. Expression of genes related to T cells and immune suppression after CpG treatment. CpG (30μg) was subcutaneously administered to C57BL/6 mice one day before 1×10^6 LLC1 cells inoculation. RNAs were extracted from days 7, 14 and 21 tumors and subjected to RNA-Seq. The expressions of genes related to T cells (A) and immune suppression (B) were shown.

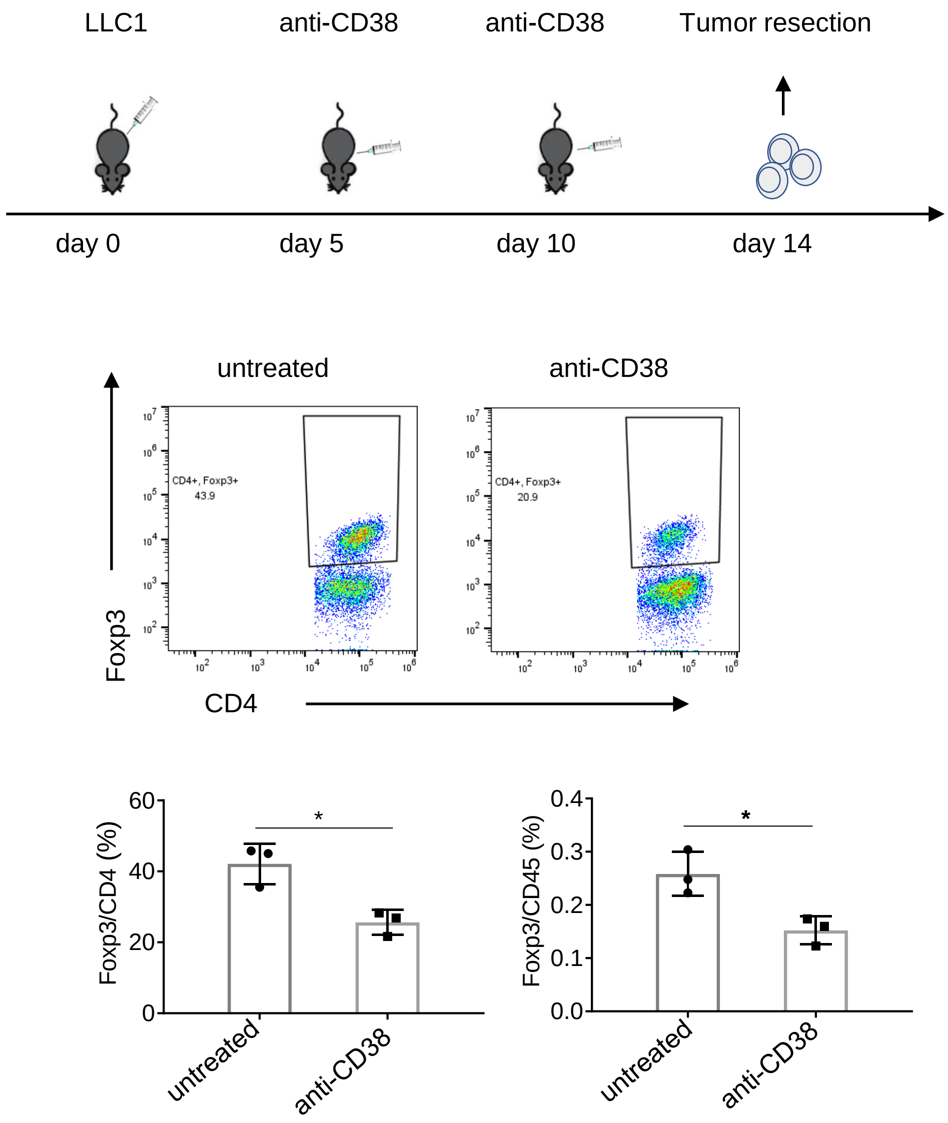

Supplementary Figure S3. Anti-CD38 mAb treatment decreased the amount of intra-tumoral regulatory T cells. Mice (n=3) were subcutaneously inoculated with 5×10^5 LLC1 cells on day 0. The mice received anti-CD38 mAb on days 5 and 10. On day 14, tumor-infiltrating cells were extracted from these mice and subjected to flow cytometry.
